# Cannabidiol in Treatment of Autism Spectrum Disorder: A Case Study

**DOI:** 10.7759/cureus.28442

**Published:** 2022-08-26

**Authors:** Lucy Ma, Sofia Platnick, Howard Platnick

**Affiliations:** 1 Engineering Science, University of Toronto, Toronto, CAN; 2 Health Sciences, Wilfrid Laurier University, Toronto, CAN; 3 Family Medicine/Chronic Pain and Medical Cannabis, Private Practice, Toronto, CAN

**Keywords:** psychiatry, child and adolescent psychiatry, pediatrics neurology, alternate therapy, cannabis (marijuana), tetrahydrocannabinol (thc), cannabidiol (cbd), adolescent cannabis use, autism spectrum disorder (asd), autism spectrum disorder and anxiety disorder

## Abstract

This case study aims to demonstrate the use of cannabidiol (CBD) with low-dose tetrahydrocannabinol (THC) in managing symptoms associated with autism spectrum disorder (ASD) to increase the overall quality of life for these individuals and their families. ASD is a neurodevelopmental disorder affecting cognitive development, behavior, social communication, and motor skills. Despite the increasing awareness of ASD, there is still a lack of safe and effective treatment options. The study includes a nine-year-old male patient who was diagnosed with nonverbal ASD. He exhibited emotional outbursts, inappropriate behaviors, and social deficits including challenges in communicating his needs with others. Since the patient was unable to attain independence at school and at home, his condition was a significant burden to his caregivers. The patient was treated with full-spectrum high CBD and low THC oil formulation, with each milliliter containing 20 mg of CBD and <1 mg of THC. CBD oil starting dose was 0.1ml twice daily, increased every three to four days to 0.5ml twice daily. Overall, the patient experienced a reduction in negative behaviors, including violent outbursts, self-injurious behaviors, and sleep disruptions. There was an improvement in social interactions, concentration, and emotional stability. A combination of high CBD and low-dose THC oil was demonstrated to be an effective treatment option for managing symptoms associated with autism, leading to a better quality of life for both the patient and the caregivers.

## Introduction

Autism spectrum disorder (ASD) is a neurodevelopmental disorder characterized by impairments in communication, social interactions, restricted interests, and repetitive behaviors [1]. ASD is associated with medical conditions such as epilepsy and deficiency in intellectual abilities [1]. The World Health Organization (WHO) estimates that one in 100 children is diagnosed with autism worldwide [2]. However, the epidemiology can vary greatly depending on geographic location. For example, the Public Health Agency of Canada reports ASD prevalence of one in every 50 Canadian children [3].

The WHO Quality of Life Instrument, Short Form (WHOQOL-BREF) developed by the WHO to effectively measure the experience of individuals with ASD, has shown that associated medical conditions negatively impact the individual’s quality of life [4]. Those with ASD, as well as their families, experience a great deal of stress and disruption with this diagnosis. Most individuals with autism will not live independently or become employable; the majority of individuals will require lifelong, primary support [5]. Families experience ongoing challenges that can affect the quality of life, including health-related factors, financial barriers, number of children, and parental stress [5]. More than 70% of those with autism suffer from comorbid conditions, most commonly anxiety, depression, and attention deficit and hyperactivity disorder (ADHD) [6]. Researchers have explored both genetic and environmental factors that influence the development of the brain and its correlation with ASD risks, such as exposure to lead, ethyl alcohol, and methyl mercury [7]. 

The objective of this case study is to demonstrate the use of cannabidiol (CBD) in a full-spectrum formulation, with low-dose tetrahydrocannabinol (THC), as a treatment option for managing symptoms associated with ASD to increase the overall quality of life for these individuals and their families. The patient in the case presented resides in Canada, where both medical and recreational cannabis are legal in all forms.

## Case presentation

The nine-year-old patient (weight 39 kilograms) was diagnosed with nonverbal ASD when he was three years old and required 24-hour supervision. He had a comorbid diagnosis of insulin-dependent diabetes mellitus (Type I diabetes), which required daily insulin injections. The patient had mild asthma with infrequent use of a salbutamol puffer. Otherwise, he was on no other medications. Due to cultural and personal preferences, the patient's mother declined to use psychiatric medications.

Prior to initiating CBD treatment, the patient exhibited behavioral symptoms with outbursts of anger and physical aggression (punching, kicking, biting, head-butting, and scratching). He required daily insulin injections, which were accompanied by self-injurious actions including head and chest punching. The patient displayed inappropriate behaviors such as playing with feces and rocking on the floor for self-soothing (stimming). He was constantly frustrated by misunderstandings when interacting with others, as he was unable to express his needs verbally. He had difficulty with sleep initiation, taking one to four hours to fall asleep and sleeping a total of four to five hours a night with frequent awakening. He required pull-up diapers each night due to incontinence. The patient attended public school with support and struggled to perform well academically. There were difficulties interacting with the teachers and other students, and following rules. 

The patient began CBD treatments through a medical cannabis clinic at age 7.5 years starting with full-spectrum high CBD and low THC oil formulation. The carrier oil is a medium chain triglyceride (MCT) coconut extract, an industry-standard formulation [8]. Each milliliter has 20 mg of CBD and < 1 mg of THC. The starting dose was 0.1 ml two times daily with meals and this was increased every three to four days until it reached a therapeutic response or 0.5ml two times a day.

Within the first two weeks of starting treatment, the patient was able to fall asleep in 10-15 minutes and sleep for 8-10 hours. He stopped wearing pull-up diapers as he was able to go to the washroom, wash his hands, and go back to bed without supervision, demonstrating a new behavior. There was reduced anxiety contributing to improved mood and concentration. He was able to practise gripping his pencil and trace letters. He started to follow simple instructions, such as retrieving three separate clothing items. At school, the patient received report cards with better grades and experienced less anger. This improvement allowed him to interact with his peers without signs of aggression. The patient's mother stated, “Since starting CBD, teachers and (the) principal have noted significant positive changes. He sits for over 30 minutes, holds a marker, and is focused enough to try and trace letters or numbers. The change has been amazing for us to witness.”

After initiating CBD, there was a significant reduction in overeating and “grazing” as the patient was content with regular meal intervals. His weight did not change significantly, aside from the expected increase with maturation (current weight 52 kilograms). Self-injurious and violent behaviors diminished with treatment, which allowed for easier administration of his daily insulin injections. Table 1 provides a comparison between the patient’s behaviors and characteristics before and after initiating CBD treatment.

**Table 1:** A comparison of characteristics before and after initiating CBD treatment for the patient in the case presented *(normal HbA1C < 5.7% and diabetic > 6.7% with treatment goal of < 7%) CBD: cannabidiol; HbA1C: hemoglobin A1c

Prior to CBD Treatment	After CBD Treatment
HbA1C: 9-10% range*	HbA1C: 8-9% range
Self-injurious behaviors	Minimal self-injurious behaviors
Inappropriate behaviors	Reduced inappropriate behaviours
Difficulty communicating verbally	Followed simple instructions
Poor sleep time and quality (4-5 hours per night)	Improved sleep time and quality (8-10 hours per night)
Poor academic performance	Improved academic performance
2-4 hours in sensory room at school	30 minutes/day in sensory room at school
Irregular eating pattern (grazing)	Regular meal intervals

The patient did not have access to the CBD for seven days while on a family trip, and his behavior regressed to pre-treatment levels. Within 24 hours, the patient experienced insomnia. After four hours, he was able to fall asleep although it was described as intermittent and fitful. After two days, there was a reduction in verbal communication and response to verbal cues, and he stopped following simple instructions. On the third day, the patient resumed self-injurious behavior. Upon returning home and restarting treatment, his sleep was regulated within two days. In the following two days, the patient regained his verbal capacity and concentration, resulting in a decrease inself-injurious behaviors.

To match physical growth, the CBD dose was later increased to 0.5ml three times a day with continued positive results including further improvement in communication and a further decline in aggressive behaviors (the upper limit prescribed was 1 ml three times a day). The caregiver did not report any side effects with the cannabidiol treatment. 

## Discussion

Conventional medical treatments such as atypical antipsychotics and selective serotonin reuptake inhibitors are used to reduce or eliminate behavioral symptoms [9]. However, these psychotropic drugs may lead to side effects such as nephropathy, hepatopathy, and metabolic syndromes [9]. Of children with autism and behavioral symptoms, 40% do not respond well to standard treatments, which motivates researchers to search for alternative pharmacological treatments, including substances derived from Cannabis sativa [10]. The use of CBD as a pharmacological treatment has been shown to relieve spasticity, pain, sleep disorders, seizures, and anxiety [10]. CBD affects the brain by interacting with the endocannabinoid system to modulate cognition, socioemotional responses, susceptibility to seizures, and nociception [11].

The endocannabinoid system consists of two identified receptors: CB1 and CB2 [11]. THC is the major psychoactive component of the cannabis plant, which interacts with both the CB1 and CB2 receptors [12]. CB1 receptors are found most commonly in the central nervous system (CNS), where THC interacts with the CB1 receptors to modulate neuronal excitability to produce psychotropic effects or “feeling high”. CB2 receptors are found primarily in microglia and vascular elements, such as in the circulating immune cells, spleen, and peripheral nerve terminals. Together, the endocannabinoid system is able to modulate emotional responses, mood, pain levels, immune system, and social behaviors [12].

Two endogenous cannabinoids identified are N-arachidonoylethanolamine (anandamide) and two arachidonoylglycerol (2-AG) [13]. These endocannabinoids are enzymes that have the ability to activate the CB1 and CB2 receptors [11]. Anandamide is a major endocannabinoid that has reduced levels in patients with ASD [13]. CBD acts as an inhibitor of fatty acid amide hydrolase (FAAH), which can break down anandamide, thereby increasing available anandamide levels [14]. By a similar mechanism, CBD reduces MAGL-mediated degradation of 2-AG, thus increasing its availability [15]. The anxiolytic and antipsychotic effects of CBD were hypothesized to be mediated by CBD-induced accumulation of anandamide and 2-AG [16]. This is supported by research conducted on valproate-treated animal models of autism, where CBD was found to act as an inhibitor of the metabolic degradation of anandamide, which leads to the accumulation of the endocannabinoid, resulting in a reduction of social interaction deficits [10].

The evidence in this case study suggests CBD can alleviate many negative symptoms associated with autism with minimal patient side effects. Oral ingestion is the preferred route for drug delivery by patients and drug developers [17]. Successful drug delivery also depends on the individual’s physiology and the physicochemical properties of the drug, such as solubility, dissolution, stability, permeability, and metabolism. Since CBD is a highly lipophilic drug, when delivered orally in solution, it can precipitate in the gastrointestinal (GI) tract, resulting in an absorption rate slower than elimination [18]. Time to peak plasma concentration following oral delivery is slow for CBD (1-4 h), and the half-life of CBD was reported between 1.4 and 10.9 h after oromucosal spray [19]. One way to increase the oral bioavailability of CBD is to administer CBD with a high-fat and high-calorie meal, since the increased micelle formation allows the CBD to be more readily available for lymphatic transportation while inhibiting drug efflux transporter activities [17].

A study by Fletcher et al. reports the usage of extracts with a high CBD low THC ratio with average CBD doses ranging from 1.8 to 6.45 mg/kg/day, similar to the range used in this case study [18]. The routine starting dose recommended for the adult patient is 5mg of CBD-predominant cannabinoid twice daily [19]. CBD titration should include increasing the dose by 10 mg per day every two to three days until a maximum of 40mg/day is reached [19]. 

The patient and the caregiver in the case study did not report any side effects with treatment. In a clinical study with 33 children, restlessness was reported in 22% of the patients and decreased with dose adjustment [20]. In that same study, CBD was discontinued in a 13-year-old male patient with severe autism due to generalized seizures after using 5 mg sublingual CBD, and the seizures resolved after antiepileptic drug treatments [20]. There is limited pharmacology research on CBD, and the potential hazards of short and long-term use need to be further investigated. Consideration might be given for CBD use when caregivers choose to avoid traditional pharmaceuticals or failure of conservative therapy.

To improve the evidence on CBD efficacy in patients with ASD, a randomized, double-blind, and placebo-controlled clinical trial should be initiated to further research various strains of CBD-enriched cannabis extracts with different dosages and durations to investigate its safety, efficacy, and tolerability.

## Conclusions

In this case, the child patient responded positively to the introduction of CBD oil treatment with reduced negative behaviors, better sleep, and improved communication. With the increasing clinical studies on the use of cannabidiol in treating patients with mood disorders, anxiety, chronic pain conditions, and other behavioral problems, it should be considered as a treatment option in managing symptoms related to autism. In the case study presented, the child patient has shown behavioral and cognitive improvements with no side effects reported. Altogether, this study presents a case that motivates further research and clinical studies to understand the molecular mechanism of CBD as well as the dosing regimes for pediatric populations, the etiology of ASD, and how various dosing affect different demographics.
